# Unsuccessful percutaneous mechanical thrombectomy in fibrin-rich high-risk pulmonary thromboembolism

**DOI:** 10.1186/s12959-015-0060-2

**Published:** 2015-09-16

**Authors:** Jernej Vidmar, Igor Serša, Eduard Kralj, Peter Popovič

**Affiliations:** Institute of Physiology, Medical Faculty, University of Ljubljana, Zaloska cesta 4, 1000 Ljubljana, Slovenia; Jožef Stefan Institute, Ljubljana, Slovenia; Institute of Forensic Medicine, Medical Faculty, University of Ljubljana, Ljubljana, Slovenia; Institue of Radiology, University Medical Centre Ljubljana, Ljubljana, Slovenia

**Keywords:** High-risk pulmonary thromboembolism, Fibrin-rich thromboemboli, Percutaneous mechanical thrombectomy, Open embolectomy

## Abstract

**Background:**

We report a case of unsuccessful percutaneous mechanical thrombectomy in treatment of a high-risk pulmonary embolism (PE). Pulmonary thromboemboli are commonly expected as a homogenous mass, rich with red blood cell content, which respond well to percutaneous mechanical thrombectomy (PMT). Catheter-based approach or surgical embolectomy are two treatment options that are usually considered for treatment of high-risk PE when the thrombolytic therapy fails or it is contraindicated due to a patient’s persisting hemodynamic compromise. Currently, selection criteria for PE treatment options are based mostly on the assessment of patient’s history. The aim of this report is to highlight a possible treatment complication in PMT of structurally heterogeneous thrombotic mass due to PMT inadequacy.

**Case presentation:**

A 32 year-old male with polytrauma was admitted to an intensive care unit after a right-sided nephrectomy and evacuation of retroperitoneal hematoma. The patient initial haemostatic disorder was improved by administration of blood preparations, an anti-fibrinolytic agent and concentrates of fibrinogen. On the third day he presented sudden onset of hemodynamic instability and was incapable of standard CTA diagnostic procedure. Urgent and relevant investigations including transthoracic and transesophageal echocardiogram confirmed a high-risk PE. PMT was performed due to contraindications for systemic thrombolysis. Long-term PMT was attempted using aspiration with several devices. No major improvement was achieved in any of the treatments and the patient died. Autopsy confirmed a large heterogeneous thrombotic mass in the pulmonary trunk folding to the right main artery. Additional histological analysis revealed a high fibrin-rich content in the peripheral surroundings of the thrombus.

**Conclusion:**

In the case, it was confirmed that the outcome of PMT was directly influenced by mechanical and histological features of the thromboembolus in high-risk PE. Formation of a rather complex thromboembolus in high-risk PE favors surgical embolectomy as the only life-saving treatment option. Current diagnostic imaging techniques do not enable precise assessment of thrombi structure and are therefore unable to identify patients who might benefit from PMT or open surgical embolectomy. Surgical backup treatment should be considered if there are no contraindications in the event of a failed catheter intervention.

## Background

An acute massive pulmonary thromboembolism, i.e., high risk PE, represents the most severe form of pulmonary embolism with mortality rates exceeding 20% irrespective of treatment [1, 2]. High-risk PE can ultimately result in sudden death secondary to massive obstruction of the pulmonary bed (approximately 10% of PE cases). Clinical presentation of high-risk PE is associated with hemodynamic instability, persistent hypotension (with hypotension defined as a sudden fall in systolic blood pressure to <90 mmHg or more, or by ≥40 mmHg from baseline) and cardiogenic shock [3, 4]. Standard management of PE involves anticoagulant treatment, though systemic thrombolysis is considered as a treatment of choice in cases of worsening cardiovascular instability or rapid respiratory failure [5–7]. Catheter-based approaches or open surgical embolectomy are usually considered when the thrombolytic therapy fails or it is contraindicated and a patient has persisting hemodynamic compromise [8–11]. PMT involves use of certain mechanical devices, ultrasound, pressurized saline injection or suction to aspirate fragments of macerated emboli following other thrombectomy techniques [12–16]. Mechanical thrombectomy exposes a thrombus surface area after fragmentation and enables local intra-clot thrombolytic agents to augment thrombolysis [17–24]. Although thrombolysis and PMT facilitate rapid improvement in haemodynamics, serious complications may occur, including bleeding, perforation or dissection of cardiovascular structures as well as failure to respond to initial immediate management [25]. Currently, selection criteria for PE treatment options, i.e., systemic thrombolysis, PMT or open surgical embolectomy, are based mostly on the assessment of patient’s history.

The structure of thrombi may influence the outcome of their resolution significantly [26]. Commonly, pulmonary thromboemboli are composed of a fibrin meshwork with entrapped RBCs and an extracellular space filled with serum, while platelets regroup into tight aggregates mimicking a solid tissue structure, rich with cellular proteins [27]. Histological characteristics of thromboemboli may vary significantly ranging from a homogenous to more complex and compacted thrombotic mass, primarily due to various etiopathogenesis of PE [28]. Contrary to the established knowledge that acutely formed thrombi are composed mostly of RBC-rich regions, rather complex histological characteristics were found in approximately 75% of mechanically retrieved thrombi [29]. There is still lack of studies, which would correlate thrombolytic or mechanical management of high-risk PE with histological characteristics of occluding thromboemboli.

The aim of this case report on PMT of the high-risk PE is to highlight a correlation between histological characteristics of fibrin-rich thrombi and the performance of PMT.

## Case presentation

A 32 year-old male was admitted to an intensive care unit after right-sided nephrectomy, evacuation of retroperitoneal hematoma and removal of tamponade from the abdominal cavity. The patient previously suffered from polytrauma in the chest, abdomen and right hip. Whole body CT excluded intracranial injury, however, it showed a contusion of the right lung, a major laceration of the right kidney, minor laceration of the right liver as well as an extensive retroperitoneal hematoma along with fractures of the left clavicle, first left rib and right iliac crest. At admission the patient was sedated, analgesied and hemodynamically stable. After the consultation with urologist and abdominal surgeon CT angiography and embolization of the right renal artery were carried out first. Subsequently, emergency surgery was performed with removal of large retroperitoneal coagulum and right-sided nephrectomy. Major bleeding occurred from the vein cava inferior shortly after the removal of retroperitoneal coagulum suggesting some vessel damage during the accident. In order to stop the bleeding and to perform haemostasis, the vein had to be temporarily compressed. The patient was hemodynamically stable at all times, however, due to major bleeding, blood preparations were administered. He received totally 4 units of packed RBCs, 5 units of fresh frozen plasma, 1 unit of platelets, 2 g of Hemocoplettan (CSL Behring, Germany), 1 g of transexsamic acid and 1500 ml of colloids. About 600 ml of blood volume was returned along with the use of Cell Saver (Haemonetics, USA). The total blood loss was estimated to be at least 2000 ml. Shortly after the surgery the patient needed transfusions of fresh frozen plasma and clotting factors as well as packed RBCs due to continuation of the haemostatic disorder with thrombocytopenia and prolonged aPTT. Laboratory screening assays performed in the next two days showed persistent thrombocytopenia, high-level of fibrinogen with a strong increase of D-dimer, prolonged aPTT and a low level of antithrombin. After the first two days, he was translated to spontaneous breathing and eventually extubated. On the third day, he presented sudden onset of hemodynamic instability, therefore incapable of standard CTA diagnostic evaluation. Urgent and relevant transthoracic and transesophageal echocardiograms were performed, which showed a massive thrombus in the pulmonary trunk along with the straining of the right ventricle.

### Percutaneous mechanical thrombectomy

PMT was performed due to its prompt availability and patient’s contraindications for systemic thrombolysis. Intervention was performed under general anesthesia through the right transfemoral catheterization with an insertion of a 10 F leading catheter. Angiography demonstrated an extensive thrombotic mass in the pulmonary trunk, expanding to the right basal and segmental arteries, however, no major defect was detected in the left pulmonary artery. Long-term PMT was attempted, for approximately 70 min in total, using aspiration with mechanical thrombectomy devices Aspirex 10 F, 6 F (Straub Medical AG, Switzerland) and Rotarex 6 F (Straub Medical AG, Switzerland), manual aspiration, balloon dilatation and fragmentation with a pigtail. During the intervention 10.000 units of heparin was administered. No major improvement was achieved and the patient became hemodynamically unstable. Local intra-clot administration of 15 mg of Actylise (Boehringer Ingelheim, Germany) was applied at the beginning of resuscitation, however, without any significant improvement and the patient died.

### Histology

Pulmonary thromboembolus was acquired within 48 h after the unsuccessful PMT procedure in routine autopsy where high-risk PE was confirmed as the main cause of death. Three major samples obtained from a large thromboembolic mass were carefully removed from the surrounding tissue and rinsed with isotonic saline of 0.9% w/v of NaCl, pH 7.4. The same samples were subsequently fixated in 10% buffered formalin for 48 h. After the fixation they were cut longitudinally and embedded in paraffin. Serial 5 μm thick cross-sections were cut from each sample. Pathologic features were initially estimated using histological sections stained by haematoxylin-eosin (HE), while an acid Picro-Mallory (PM) stain was used to reveal fibrin layers (clear red), RBCs (yellow) and the presence of connective tissue (blue). Subsequently, the sections were immunohistochemically stained against RBCs (GPA), platelet components (CD61) and fibrin using monoclonal antibodies (Anti-Human Glycophorin A, β-3 integrin Anti-Human CD61 and Polyclonal Rabbit Anti-Human Fibrinogen, DakoCytomation, Denmark). Monoclonal mouse anti-human antibodies against Col I (Sigma Aldrich) were used additionally to visualize a possible presence of collagen inclusions in the thromboembolus. Immunohistochemical staining was performed using a Ventana Bench-Mark automatic stainer with a streptavidin-biotin peroxidase complex and diaminobenzidinetetrahydrochloride as a chromogen.

## Discussion

PMT techniques (aspiration, fragmentation and rheolytic thrombectomy) are designed to optimize target-vessel recanalization and to shorten the procedure time in large vessels or bypass grafts. Specifically, in high-risk PE the rationale behind PMT is a rapid relief of a central pulmonary occlusion. In our case, the angiographic outcome of used PMT devices was strongly influenced by the histological characteristics of the occluding thromboembolus. Massive occlusions demonstrate extremely low recanalization rates as well as long recanalization times without any significant improvement. Most plausible explanation why the morphology of the thrombus influence the outcome of the initiated PMT is most likely a high-protein (fibrin-rich) content in a structurally heterogeneous thrombotic mass. This observation could be supported by the results of autopsy, histology and retrospective analysis of laboratory findings.

**Fig. 1:**
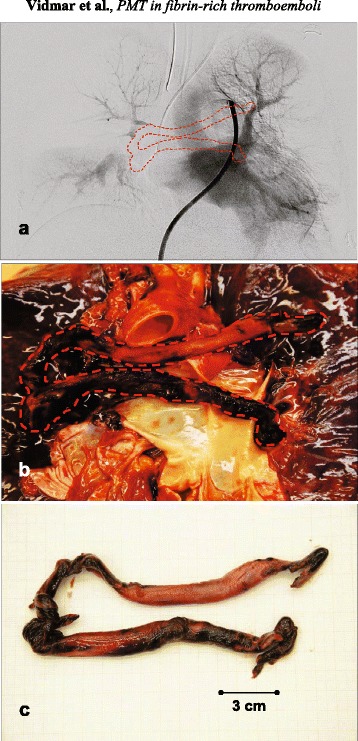
Selective pulmonary angiogram (**a**) revealing massive pulmonary thrombus (encircled symbolically in red line) causing a central obstruction in the pulmonary trunk and in the right main pulmonary artery. Marcophoto images (**b**, **c**) obtained during autopsy confirming the presence of a large heterogeneous thrombotic mass in the pulmonary trunk folding to the right pulmonary artery

**Fig. 2:**
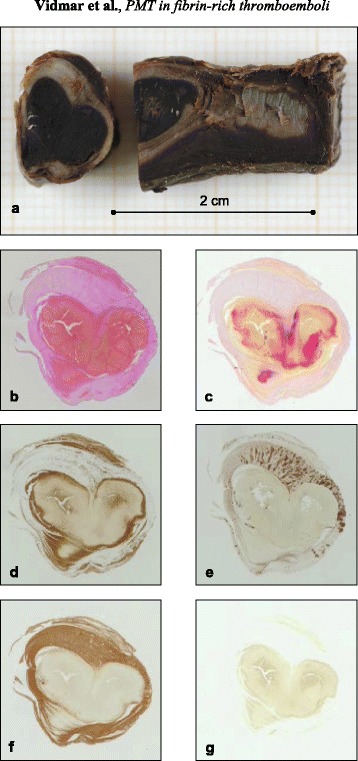
Gross morphology of a formaldehyde-fixated representative sample of the heterogeneous thromboembolus (**a**). Histological analysis in a cross-section of the same representative sample using non-specific haematoxylin-eosin staining (**b**) and Picro-Mallory ((**c**), RBC-rich regions appear yellow and fibrin clear red). Immunospecific staining for glycophorin A ((**d**), RBCs appear brown), anti β-3 integrin ((**e**), platelets appear brown), anti-fibrin ((**f**), fibrin meshwork appears brown) and anti-collagen ((**g**), no true positive reaction). Original magnification: 2×

The autopsy (Fig. 1b and c) confirmed our assumptions that the occlusion of the pulmonary trunk along with the right pulmonary artery was caused by a large, highly heterogeneous, twisted and folded thrombotic mass. Furthermore, the autopsy excluded any sign of a leading catheter damage, which could suggest a PMT malfunction. Additionally it excluded any signs of vein cava inferior wall trauma, which could be caused due to surgically performed haemostasis and could represent a possible origin for thrombus formation. Macroscopic cross-sections of representative samples of the thromboembolus showed thick white protein laminations, concentrated mainly in the peripheral surrounding, while compacted RBC-rich regions were accumulated in the central part of the thromboembolus. Histological analysis of the thromboembolus (Fig. 2) using conventional non-specific HE, semi-specific trichrome PM and immunospecific staining for GPA, CD61 and fibrin confirmed that the high RBC content were found mainly in central parts of the thromboembolus (yellow regions in Fig. 2c and brown in Fig. 2d). On the contrary, large amounts of a dense fibrin meshwork interspersed with moderate platelet areas were found in peripheral regions of the thromboembolus (brown in Fig. 2e and Fig. 2f). Histological and immunohistochemical analysis revealed no signs of early organization as well as no sign of large collagen inclusions, which suggests that the formation of thromboembolic impact occurred rather fast, i.e., in less than three days [30].

The criteria for using PMT were in our case restricted due to the patient’s presenting in hemodynamic shock and contraindications for systemic thrombolysis. Several PMT devices such as Aspirex, Rotarex, manual aspiration, balloon dilatation and pigtail were used in attempts of recanalization, however, all of them failed. One of the reasons for that is a large accumulations of fibrin and cellular components, which made the thromboembolus stiff/hard. The stiffness of the fibrin-rich thromboembolus originates from a substantial fibrin meshwork that reduced the performance of PMT devices [31]. This was also shown in a study by Yuki et al. [29]. Furthermore, the unusual properties of the fibrin-rich thromboembolus (stiffness/noncompliance) can also explain why the thromboembolus was less susceptible to fragmentation, thus preventing piecemeal thrombectomy. In a case of partial fragmentation, it is expected that the captured parts would not pass through the guiding catheter tip due to their size and mechanical properties.

There is currently no widely accepted protocol for PMT, however, a systematic review analyzing a variety of PMT methods showed that they were all generally safe and effective with no significant difference among the methods and with low complication rates [32]. Up to now, only a few cases of failed PMT are found in the literature. Most of them are associated with chronic PE resulting from multiple embolic events or due to the presence of organized and adherent thrombi [33].

Etiopathogenesis of the rather complex fibrin-rich thromboembolus could be attributed to a combination of patient’s initial underlying disorder, i.e. politrauma with large blood loss, following necessary surgery and subsequently administrations of fibrinogen, blood transfusions and anti-fibrinolytic drug. Laboratory screening assays in the follow-up are in accordance with persistent changes in the patient’s haemostatic functions such as thrombocytopenia, strong increase in D-dimer and prolonged aPTT. Results of the laboratory tests suggest that pathogenesis of DIC-associated disorder might have occurred with a predominating thrombotic component, favoring formation of the fibrin-rich thromboembolus.

It is rather unlikely that the structure of the thromboembolus was changed during the PMT procedure and attempts of intra-clot lysis. Our observation is supported with a post-mortem histological and immunohistochemical analysis of the occluding thromboembolus. The analysis revealed no signs of early organization as well as no signs of large collagen inclusions, along with a fibrin-rich content. This suggests that the formation and propagation of the thromboembolus occurred rather fast, i.e., in less than three days, with a massive fibrinogen consumption.

Other than PMT, we found no major hemodynamic improvement after the introduction of intra-clot administration of fibrin-specific thrombolytic agent. This observation is in agreement with results of *in vitro* fibrinolysis, which suggest that dissolution of the fibrin-rich clots proceeds at a rather slow rate due to an impeded progression of the local thrombolytic agent [34].

Current standard diagnostic imaging techniques do not enable precise histological characterization of thrombi *in situ*. As a result of that two different thromboemboli may show an identical angiographical profile, however they would respond quite differently to PMT due to their relevant properties that remained angiographically undetected. On the other hand, advanced multiparametric MRI already enables discrimination between cell-rich or protein-rich components of thrombi [35]. Translation of advanced MR imaging techniques from an experimental *in vitro* to clinical *in vivo* environment, such as, free-breathing MRI techniques, multiparametric MRI acquisition and application of fibrin-specific MRI contrast agents could already enable a more precise morphological and functional assessment of thrombi and therefore ease treatment decision making, i.e. PMT vs. surgical embolectomy [36–38]. Specifically, in our case, a prompt use of advanced imaging diagnostic tools, for example multiparametric MRI, could enable right treatment decision so that the patient would be treated by surgical embolectomy instead by PMT. Nevertheless, a multidisciplinary approach is desired to determine the best treatment for patients with life-threatening PE. In the event of a failed catheter intervention, surgical backup treatment should be considered if there are no contraindications.

## Conclusion

We conclude that mechanical and histological features of thromboembolus may directly influence the outcome of PMT as shown in presented case report. Specifically, in critically-ill patients formation of rather complex thrombi may occur, favoring surgical embolectomy as the only life-saving treatment option. Currently diagnostic imaging lacks of more price assessment of thrombi structure. Therefore, it is difficult to identify patients between the two treatment options, PMT or surgical embolectomy. In the event of a failed PMT surgical backup should be considered if there are no contraindications.

## Consent

Written informed consent was obtained from the patient’s relative for publication of this Case report and any accompanying images. A copy of the written consent is available for review by the Editor-in-Chief of this journal.

## Abbreviations

aPTT: Activated partial thromboplastin time
DIC: Disseminated intravascular coagulation
F: French
PE: Pulmonary embolism
PMT: Percutaneous mechanical thrombectomy
RBC: Red blood cell
